# Cloud-based solution to identify statistically significant MS peaks differentiating sample categories

**DOI:** 10.1186/1756-0500-6-109

**Published:** 2013-03-23

**Authors:** Jun Ji, Jeffrey Ling, Helen Jiang, Qiaojun Wen, John C Whitin, Lu Tian, Harvey J Cohen, Xuefeng B Ling

**Affiliations:** 1 Departments of Surgery, Stanford University, Stanford, CA 94305, USA; 2 Departments of Pediatrics, Stanford University, Stanford, CA 94305, USA; 3 Departments of Health Research & Policy, Stanford University, Stanford, CA 94305, USA; 4 State Key Laboratory of Industrial Control Technology, Institute of Industrial Process Control, Zhejiang University, Hangzhou 310027, China

## Abstract

**Background:**

Mass spectrometry (MS) has evolved to become the primary high throughput tool for proteomics based biomarker discovery. Until now, multiple challenges in protein MS data analysis remain: large-scale and complex data set management; MS peak identification, indexing; and high dimensional peak differential analysis with the concurrent statistical tests based false discovery rate (FDR). “Turnkey” solutions are needed for biomarker investigations to rapidly process MS data sets to identify statistically significant peaks for subsequent validation.

**Findings:**

Here we present an efficient and effective solution, which provides experimental biologists easy access to “cloud” computing capabilities to analyze MS data. The web portal can be accessed at http://transmed.stanford.edu/ssa/.

**Conclusions:**

Presented web application supplies large scale MS data online uploading and analysis with a simple user interface. This bioinformatic tool will facilitate the discovery of the potential protein biomarkers using MS.

## Findings

### Background

Discovering potential protein biomarkers using mass spectrometry (MS) is both promising and challenging in high-throughput biology[1]. Among the proteomic profiling techniques, differential protein abundance comparison analysis across samples is an important one. Most previous MS based peak detection approaches lack the integration of discriminating feature selection (MS peaks) tools with methods to determine which proteins are appropriate for validation analyses. Moreover, it has been difficult for experimental biologists to configure MS analytic tools, which usually contain many user-defined parameters. We have developed simultaneous spectrum analysis (SSA) [2] for effective and efficient MS peak selection. SSA uses only two key parameters, one of which is to locate peaks in the MS spectra and the other is to set quality thresholds to select robust features. Compared with the other existing methods, SSA improves the number and quality of lower signal intensity peaks. In addition, SSA is less likely to introduce systematic bias when normalizing spectra. Subsequent to feature selection, false discovery rate (global or local FDR) analyses (gFDR [3]; lFDR [4,5]), which can simultaneously analyze vast number of features, needs to be applied for high dimensional peak differential analysis. Therefore, integration of SSA and FDR methods can be an effective approach for MS peak analyses between sample populations.

However, there are practical difficulties in the integration of SSA-FDR methods. Each method usually requires difficult computations. Another challenge is that the volume of data of a typical MS analysis task includes hundreds of spectra files and easily exceeds 100 megabytes of content. To address these issues, a cloud based web portal was developed to easily upload large MS data sets, select reproducible features (peaks), identify statistically significant peaks, correct for multiple hypotheses in order to determine which differentially expressed proteins are worth pursuing for subsequent biomarker validations quickly, and dynamically generate graphic output for meaningful interpretation.

## Implementation

**Figure 1:**
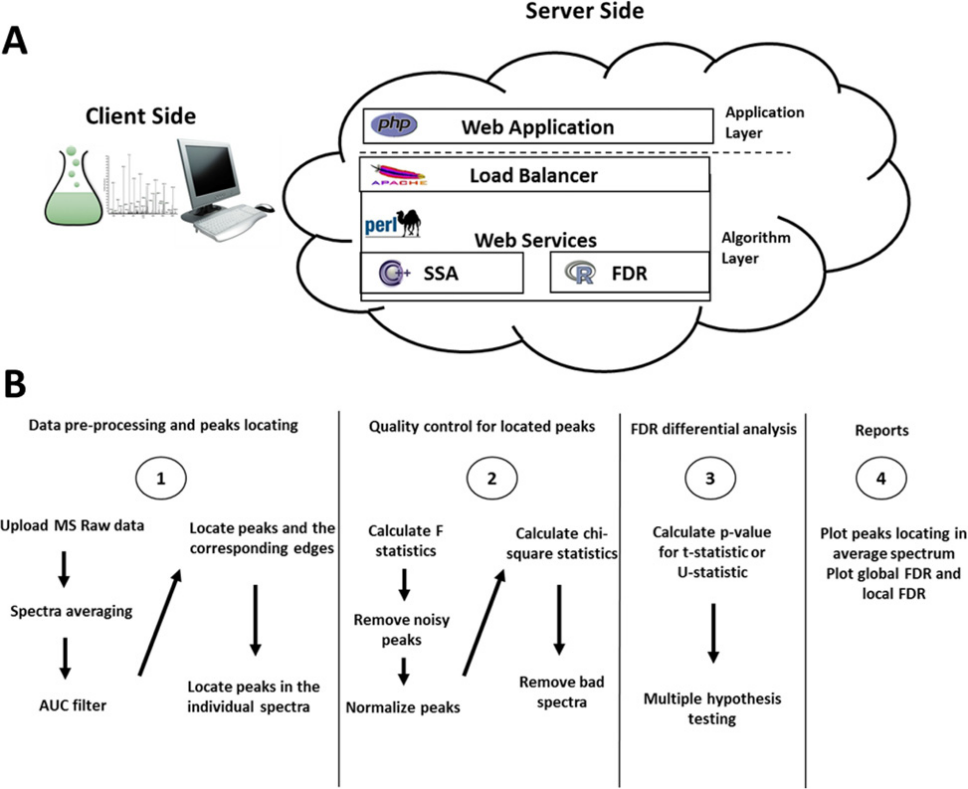
**Online MS data analysis solution. (A)** Schematic diagram. **(B)** Data analysis pipeline diagram.

The web portal is built on Apache (http://www.apache.org/). PHP (http://www.php.net/) is used to build the simple front end, while Perl (http://www.perl.org/) and R (http://www.r-project.org/) are used to implement the web service functionalities. The design schematic drawing is illustrated in Figure 1A. Typical web services use Simple Object Access Protocol (SOAP) or Representational State Transfer (REST). Our web service is simplified with Common Gateway Interface (CGI) implementation. The algorithms were wrapped into web service with CGI application so that the algorithms can be invoked by any platform following the standard of the interface. By design, our computing algorithms can easily be updated without changing the web services interfaces. The algorithms and their black-box web services can be integrated by third party web building scripts.

With large mass spectrometry data, any online MS analytical tool requires multiple files to be uploaded in parallel to be a practical solution. Also the MS data could be very large which is very hard to upload via http protocol. To handle these problems, the Uploadify module (http://www.uploadify.com/) was integrated to allow large MS raw data to be uploaded efficiently. Uploadify is a jQuery(http://www.jquery.com/) plugin which supports multiple file upload functionality to the website. It has HTML5 and Flash versions, which support all common used web browsers well. Using R and perl, the algorithm functionalities are developed as web services for web-based applications. SSA-based peak finding, alignment and indexing method was effectively integrated with FDR analysis for differential feature discovery. The analysis results are summarized as graphs, excel tables, and text files, that can readily be downloaded from the server as a zipped package.

To demonstrate the efficiency and effectiveness of the web portal, an MS data set with 135 megabytes of data, available online for download, is included as a demonstration example. The data include 202 spectra, each of which is recorded in a comma-separated file, and one metadata file describing the spectra data. The data file analysis was performed on a laptop with 4 gigabyte memory and Intel Core i5-2467M 1.6GHz CPU. It requires only 21 seconds to upload all files in the MS data set. An additional 40 seconds was required to complete the subsequent calculation as well as the report with graphs and tables.

## Results and discussion

The web site mainly includes two applications: (i) common peak discovery across spectra using SSA; (ii) differential analysis of indexed peaks and FDR correction. Detailed instructions can be found on the web site. The applications can be applied to high-throughput MS data analysis with large sample sizes. The flowchart of the application is shown in Figure 1B. Both the spectra and the metadata are uploaded as MS raw data. After data upload, the MS data are processed with SSA for peak detection.

**Figure 2:**
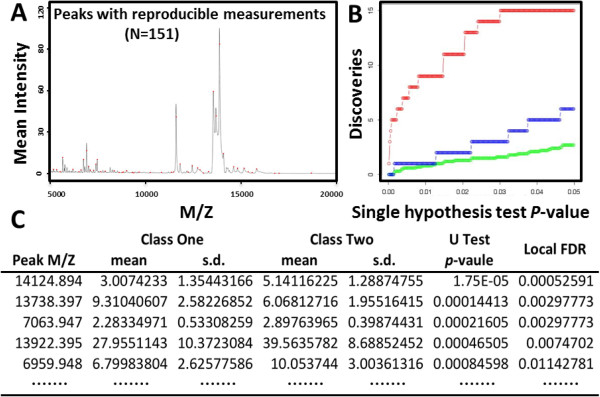
**MS analysis results. (A)** Indexed peaks (red dots) extracted from all assayed samples by SSA algorithm. **(B)** Global false discovery analysis. The horizontal axis represents the p-value thresholds and the vertical axis shows the number of discoveries. The red, green and blue lines represent total discoveries, average and the 95 percentile of the false discoveries. **(C)** lFDR output. s.d.: MS peak signal standard deviation.

SSA can discover MS peaks across MS data sets. At first, SSA maps spectra onto a uniform m/z axis using linear interpolation. Then, a composite spectrum is generated in a two-step process by averaging the average spectrum of each group (in order to give equal weight to each group). To detect the peak location, an area under the curve (AUC) filter is applied to the composite spectrum. Each local maximum in an AUC-filtered composite spectrum is recorded as a peak location, and the peak edges are located as well. Then, the AUC filter is applied to each spectrum to find and quantify the peaks, using the peak edges determined previously. To remove the noisy peaks (peaks that are poorly reproducible between replicates), an F-test is applied on peak signal content, with a confidence threshold of 95-99%. The threshold can be adjusted to 80-90% for large-scale protein profiling. The detected peaks are normalized with expectation-maximization (EM) algorithm [6] to determine the scale factors for peak normalization. A chi-squared(*χ*^2^) statistic is calculated for each spectrum to discard the bad spectra. There are several parameters with default values for fine-tuning SSA results: the *min.peak.widths*, *max.peak.widths*, *peakWidthSteps*, *m.z.regions*, *m.z.step* are used to control the locating of peaks, while the *F.test.threshold* and *chi.sq.threshold* are used to remove the noisy peaks and spectra with poor quality, respectively. Tutorials of the parameters can be found online at the web site. SSA-discovered MS peaks are shown in Figure 2A where the red dots on the composite graph indicate the peaks.

The resulting peak list is then subjected to differential test analysis with t or U test that assigns p-values to each MS peak comparing the groups. Multiple hypotheses testing of features (protein MS peaks) is addressed by the subsequent FDR analysis. The total discoveries count features with Student’s t-test or Mann-Whitney U-test p-value lower than a predefined threshold. Thresholds of single feature test p-value can be surveyed comprehensively to reveal total or false discoveries to calculate gFDR. To rank the MS peaks, the lFDR assigns significance measures to each feature. The analysis results are summarized in graph (gFDR, Figure 2B) and table (lFDR, Figure 2C) forms. The users can get FDR at different levels by manipulating the downloadable excel files.

## Conclusions

Our MS analysis portal is mainly designed for experimental biologists to process MS data sets and compare protein abundance to discover biomarkers. The analysis begins with a raw data upload and ends with a set of data sheets and graphs for easy data presentation and visualization. With all the functions provided by the Stanford University’s computing cloud, no informatics knowledge is required for the end users and all the analysis results, including graphs, Excel and text files can be downloaded from the web site. The website works best when the uploaded spectra represent the entire sample (e.g. unfractionated plasma, serum, CSF). For fractionated samples, the downloaded results files for each fraction can be compiled into a single file representing all the results and submitted to the FDR website. With the computational capacity guaranteed by cloud-based computing, the end users can access the server, and get the computing results rapidly and accurately. This cloud algorithm integration should be of general interest to those working in the field of high-throughput proteomics-based biomarker discovery.

## Availability and requirements

The website can be accessed using any major browsers as follows: Webserver: http://transmed.stanford.edu/ ssa/Sample MS data: http://transmed.stanford.edu/ssa/sample.data/3277_pH.12_CM10_1170005079_F_3400.0. csvSampe meta data: http://transmed.stanford.edu/ssa/sample.data/metadata.txt Site help: http://transmed.stanford.edu/ssa/ssa_analysis.ppt

## Competing interests

The authors declare that they have no competing interests.

## Authors’ contributions

JJ designed the solution infrastructure, implemented the mainframe of the web portal, and revised the manuscript. JL assisted with the infrastructure design and implemented the SSA functionality of the solution. HJ assisted with the infrastructure design and implemented the uploading functionality of the solution. QW assisted with the infrastructure design, drafted the initial manuscript and revised the manuscript. JCW assisted with the features design and revised the manuscript. LT assisted with the infrastructure and features design. HJC conceptualized the study and revised the manuscript. XBL designed the study and revised the manuscript. All authors read and approved the final manuscript.
